# Sex Difference in MasR Expression and Functions in the Renal System

**DOI:** 10.1155/2022/1327839

**Published:** 2022-09-10

**Authors:** Samira Choopani, Mehdi Nematbakhsh

**Affiliations:** ^1^Department of Physiology, Isfahan University of Medical Sciences, Isfahan, Iran; ^2^Water & Electrolytes Research Center, Isfahan University of Medical Sciences, Isfahan, Iran

## Abstract

Renin-angiotensin system (RAS), as a critical system for controlling body fluid and hemostasis, contains peptides and receptors, including angiotensin 1-7 (Ang 1-7) and Mas receptor (MasR). Ang 1-7 implements its function via MasR. Ang II is another peptide in RAS that performs its actions via two Ang II type 1 and 2 receptors (AT1R and AT2R). The functions of AT2R and MasR are very similar, and both have a vasodilation effect, while AT1R has a vasoconstriction role. MasR affects many mechanisms in the brain, heart, blood vessels, kidney, lung, endocrine, reproductive, skeletal muscle, and liver and probably acts like a paracrine hormone in these organs. The effect of Ang 1-7 in the kidney is complex according to the hydroelectrolyte status, the renal sympathetic nervous system, and the activity level of the RAS. The MasR expression and function seem more complex than Ang II receptors and have interacted with Ang II receptors and many other factors, including sex hormones. Also, pathological conditions including hypertension, diabetes, and ischemia-reperfusion could change MasR expression and function. In this review, we consider the role of sex differences in MasR expression and functions in the renal system under physiological and pathological conditions.

## 1. Introduction

**Figure 1:**
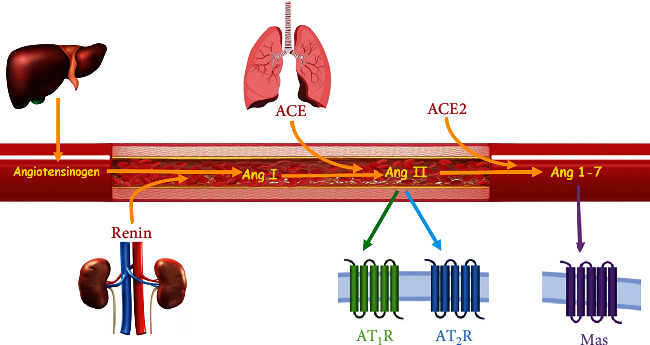
Angiotensin (Ang) 1-7 is formed from Ang II by angiotensin-converting enzyme 2 (ACE2). AT1R: angiotensin type 1 receptor; AT2R: angiotensin type 2 receptor; Mas: Ang 1-7 receptor.

The classic renin-angiotensin system (RAS) structure begins from renin secretion via juxtaglomerular cells in the kidney. Renin converts angiotensinogen to angiotensin (Ang) I. Then Ang-converting enzyme (ACE) converts Ang I to Ang II in the lungs. In parallel, ACE2 converts Ang II to Ang 1-7 (Figure 1). Evidence demonstrates that Ang 1-7 mainly plays a counterregulatory role in the RAS [1, 2]. ACE2 is a carboxypeptidase enzyme that was identified as a homolog of ACE [3]. This enzyme causes vasodilation by cleaving Ang II. Due to the use of ACE inhibitors in the treatment of hypertension and heart failure, there is considerable interest in understanding the function and expression of ACE2 in various human organs [4]. One study reported the expression pattern of ACE2 in more than 150 different cell types from 16 different organ systems based on a detailed immunohistochemical analysis. The highest expression was marked in the intestinal tract, followed by the kidney, testis, gall bladder, and heart [4]. The most critical RAS peptide is Ang II which implements its functions by two primary receptors of type 1 (AT1R) and type 2 (AT2R). AT1R and AT2R activities are not similar; while AT1R has a vasoconstriction effect, AT2R has vasodilation in the cardiovascular and renal systems [5–7]. There is well-documented research about the role of sex differences on Ang II and its receptors [8–11]. A lower AT1R/AT2R ratio was detected in females [12], and it also is noted that AT2R may be upregulated by estrogen [13]. Ang 1-7 is the other critical component of RAS which induces vasodilation via its specific receptor of Mas receptor (MasR). MasR affects many mechanisms in the brain, heart, blood vessels, kidney, lung, endocrine, reproductive, skeletal muscle, and liver [14]. Ang 1-7 probably acts like a paracrine hormone [15]. It is produced locally in the kidney, one of the essential organs for the activity of this peptide. Also, renal vessels are highly influenced by Ang 1-7 [14]. The effect of Ang 1-7 in the kidney is complex. The renal hydro electrolyte, sympathetic nervous system, and the activity level of the RAS in the kidney are some cases that affect this response [16]. Studies have shown that in isolated and intact kidneys of Wistar and Sprague-Dawley rats, infusion of Ang 1-7 causes diuresis and natriuresis [16]. Ang 1-7 has a counter-regulatory effect on AngII/AT1R pathway by reducing renal vascular resistance (RVR) and increasing renal blood flow (RBF) [6].

There are heterodimerization and functional interactions between MasR and AT1R or AT2R [14].

Previous studies indicate that treatment with RAS inhibitors increases ACE2 expression or action in numerous organs, including the heart [17–26], kidney [17, 22, 23, 27–29], aorta [30, 31], and lung [32, 33] in rodents. So, increasing Ang 1-7 via treatment ACEIs and ARBs help the antihypertension and renal protection [34]. Conversely, Wang et al. reported that inhibition of Ang II by losartan did not influence the tissue expression of ACE2 [35], as well as administration of Ang II for two weeks, increased blood pressure. However, it could not alter the ACE2 mRNA expression in several organs, including the lung, ileum, left ventricle, and kidney [35]. Their study showed that ACEIs, via reducing tissue inflammation and improving organ injury, act on ACE2 and do not directly affect ACE2 expression in vivo [35].

However, the role of sex differences in MasR expression and activity is still challenging in the literature. In this review, we consider a variety of information databases such as PubMed, Google Scholar, and Scopus to specify the role of the sex hormone estrogen on the expression and function of the Ang 1-7/MasR axis.

## 2. Mas Receptor

MasR is a G-protein recognized in 1986 during the investigation of tumors [36, 37], and later, it was known as a receptor for Ang 1-7 since 2003 [38]. This receptor stimulates the production of nitric oxide (NO) and prostaglandin (PG) in the endothelial, ovary, and coronary arteries [39–41]. It is well known that NO and PG cause vasodilatory, antiproliferative, antithrombotic, diuretic, and natriuretic properties [39, 40, 42]. MasR expresses in many body organs like the heart, kidney, vascular system, brain, testis, lung, liver, spleen, tongue, and skeletal muscles [43, 44]. MasR also could be found in the renal cortex, proximal tubule, thick ascending loop of Henle, collection duct [45], afferent arteriole, tubular epithelium, predominantly on the apical surface [46], and mesangial cells [47]. However, less information is available about its physiological function.

### 2.1. Renal MasR Expression and Sex Differences

Like other RAS receptors, the MasR expression depends on various factors and pathological conditions such as sex hormones, diabetes, hypertension, etc. MasR expression is different between the sexes in the renal system. The mRNA levels of the MasR were higher in adult female rats' kidneys than in males [48, 49]. One study found that sex chromosomes regulate AT1R, AT2R, and MasR gene expression in the renal cortex [50]. Different basal blood pressure effects on MasR expression. For example, 14-day Ang 1-7 infusion in normotensive Wistar rats did not change renal MasR expression, but conversely, in spontaneously hypertensive (SHR) rats, renal MasR expression decreased [51]. The induced hypertension also affects MasR expression depending on sex [52, 53]. Generally, it has been found that in the renal cortex of SHR models, Ang II administration increased MasR expression in females while no change was observed in males [54]. In two-kidney, one clip (2K1C) hypertensive model, MasR expression decreased [52]. Upregulation of intrarenal angiotensinogen and ACE expression and down-regulation of ACE2 and MasR gene expression contributed to obtaining a high AngII/Ang 1-7 ratio, which facilitated the development of hypertension [55]. It is also reported that in mRen2.Lewis hypertensive rats, estrogen deficiency decreased plasma Ang 1-7 levels, so the increased level of Ang II/Ang 1-7 ratio suggests that lack of estrogen leads to the dysregulation of the RAS [56, 57]. In addition, in mRen2.Lewis rats, renal Ang II was twofold higher in males than females, while Ang 1-7 level was threefold higher in females [58].

In the renal ischemia/reperfusion (I/R) model, intrarenal MasR expression increased [59, 60]. Numerous studies have shown that in diabetic mice, the expression of AT1R and AT2R proteins is upregulated, and the expression of MasR protein is downregulated [61–64]. After ARB treatment, renal AT1R expression was downregulated, and renal MasR expression was upregulated [65, 66].  Table 1 shows the MasR expression changes in different physiological and pathological conditions. In physiological conditions, the MasR expression is higher in females than males. However, in nonphysiological conditions, the higher expression of the MasR in females acts as a support arm in the body. This is probably one of the reasons why kidney and cardiovascular diseases are less common in women of reproductive age than men.

### 2.2. Renal MasR Function and Sex Differences

From a physiological point of view, the MasR function is more complex and does not have complete transparency. On the one hand, MasR is known as the Ang 1-7 specific receptor, and on the other hand, its interaction with RAS receptors created an unforeseen situation. That is difficult to accurately identify its exact function because it is affected by various parameters such as bradykinin, gender, hypertension, etc. In the following, we mention some of Ang 1-7 and MasR's effects on renal functions under several normal and pathological conditions.

In isolated renal arteries, Ang 1-7 did not affect vascular function, but following Ang II infusion, it decreased vasoconstriction [67]. In MasR knockout mice, Ang 1-7 vasodilation was reduced about % 40 [68]. Following Ang II infusion in sodium-replete Wistar rats, Ang 1-7 could not reduce RBF [69]. In water-loaded rats, Ang 1-7 injection decreased urine volume, which was reversed by MasR antagonism [70]. Dilauro and Burns indicated that Ang 1-7 and MasR are involved in regulating natriuresis [71]. In virgin female rats, administration of A779 (MasR antagonist) significantly increased urine flow, offering that Ang 1-7 caused an antidiuretic response, but in pregnant rats, the response was reversed [72]. In water-loaded mice, AVE-0991 (nonpeptide Ang 1-7 agonist) administration decreased urine volume and increased urine osmolality [73], and these effects were blocked by MasR, AT1R, and AT2R antagonism.

Cross-talk mechanisms between the MasR, AT1R, AT2R, bradykinin B2, and vasopressin V2 receptors control water transportation in the kidney [70, 71, 74, 75]. C57BL/6 Mas knockout mice revealed a renal phenotype determined by sodium and water retention, glomerular hyperfiltration, microalbuminuria, renal fibrosis, and upregulation of renal AT1R [76]. In the 2K1C model, treatment with a selective Ang 1-7 receptor blocker or an ACE2 inhibitor worsens hypertension and renal function [77]. In male SHR, Wistar-Kyoto (WKY), and streptozotocin-induced diabetic rats, Ang 1-7 promoted RBF via increasing PG and NO release [78]. Sullivan et al. documented that in male or female SHRs, A779 did not affect baseline blood pressure [54]. This result was compatible with other examinations in control and diabetic SHRs, WKY, and 2K1C male rats [77, 79, 80]. However, in female SHRs, Ang II infusion in the presence of A779 compared with the absence of A779 produced a more powerful rise in mean arterial pressure and severe proteinuria [54]. These findings confirmed that Ang 1-7 buffers rapid increase in blood pressure and renal damage in female SHRs normally [54]. Diabetic nephropathy is associated with the Ang 1-7 levels in the kidney [81]. In an experimental study, in type 2 diabetic db/db mice, Ang 1-7 treatment reduced kidney injury [82]. In the diabetic Akita mice model and the animals were treated with Ang 1-7 with or without MasR antagonist, Ang 1-7's action decreased urinary albumin/creatinine ratio, tubular apoptosis, and renal oxidative stress [83]. In another study, the results were the opposite, so the administration of Ang 1-7 in female albino Wistar rats caused an increase in oxidative stress and kidney damage [84]. Zhang et al. showed that in male Wistar rats, Ang 1-7 infusion by reducing oxidative stress and glomerular sclerosis is better than blocking angiotensin receptors to protect the kidneys [85]. Shao et al. indicated that Ang 1-7 infusion in diabetic Sprague-Dawley rats did not postpone the kidney damage [86]. Estrogen therapy in ovariectomized (OVX) rats increased vasodilation which was eliminated by Ang 1-7 receptor antagonist (D-[Ala7]-Ang 1-7) [87]. In male and female rats, Ang 1-7 infusion increased RBF, and A779 administration attenuated this response in both sexes, although this response was more considerable in females than males [88]. Sampaio et al. documented A779 decreased RBF in male rats [89]. In OVX rats, MasR blockade decreased RBF response to Ang 1-7, which was increased by estradiol treatment [90].

In male and female Wistar rats, simultaneous AT1R, AT2R, and MasR blocking, compared with only AT1R, AT2R blocking, Ang 1-7 infusion increased RBF response in males but not in female animals. So this finding indicated that renal vascular response to Ang 1-7 may not be exerted by MasR alone in males [91, 92]. Some actions of Ang 1-7 could inhibit by the co-blockade of AT1R and AT2R. For example, in a study, renal perfusion pressure (RPP) was controlled at 80 and 100 mmHg in male and female Wistar rats. AT1R, AT2R, and MasR blockade, then the pressure natriuresis and diuresis, RBF, and RVR response were studied. Data analysis shows that RBF, RVR, and the serum level of renin in both genders did not change whether MasR was blocked or not [93]. However, when the level of RPP was 100 mmHg, in the presence of MasR, urine flow rate (UF) and sodium excretion (UNaV) increased significantly in male but not female rats [93]. As a result, co-blocking AT1R and AT2R, MasR effect in pressure diuresis and natriuresis sex-dependent, and in the presence of MasR, this response increased in male but not female rats [93]. Ueda et al. investigated the interaction between Ang 1-7 and bradykinin (BK) in forearm-resistant vessels of normotensive healthy men. Their study showed that Ang 1-7 intensifies the BK vasodilatory effect, and NO had an essential role in the interaction between BK and Ang 1-7 [94]. Dehghani et al. documented that co-administration of BK and A779 increased RBF response to Ang 1-7 in ovariectomized (OVX) than OVX plus estradiol (OVX + Est) treated rats. The administration of BK alone reversed this response. So, they concluded that estradiol vasodilatory response depends on MasR existence [95]. Saberi et al. showed that in OVX female Wistar rats with estradiol therapy when bradykinin B2 receptor (B2R) was blocked, Ang 1-7 infusion increased RBF; However, co-blockades of MasR and B2R abolished this response [96]. These results show that when B2R was blocked alone, estradiol therapy could promote RBF response to Ang 1-7 infusion. However, the co-blockade of MasR and B2R decreases the role of estradiol [96]. Safari et al. documented that the co-blockade of AT2R and MasR eliminates the vasoconstriction effect of A779. Thus, AT2R activation is required for vasoconstriction effects due to MasR block [97].

There are sex differences in hemodynamics parameters to graded Ang II administration in 2K1C hypertensive rats with co-blockade AT1R and MasR. So RBF increased significantly in males than females when AT1R and MasR were blocked [98]. Also, in normotensive OVX rats, administration of estradiol and A779 reduced RBF in response to graded AngII infusion significantly, but this response was eliminated by induction of 2K1C [99]. This response confirms that MasR function is different in normotensive and hypertensive conditions.

AVE0991 treatment in the renal I/R model in male C57BL/6 Wild-type or Mas−/− mice promoted renoprotective effects and decreased infiltration of leuko­cytes in the kidney [60]. Karimi and Nematbakhsh reported that in 2K1C rats, after partial I/R, MasR has an essential role during Ang II infusion [100] because, after A779 administration, RBF response to Ang II infusion increased in males rats. In another study, after a moderate renal I/R, A779 alone or A779 + PD123319 (AT2R antagonist) infusion increased the RBF and RVR responses to Ang II infusion significantly in females but not in the male rats [101], so that simultaneous AT2R and MasR blockade decreased renal vasoconstriction following Ang II infusion. Unilateral ureteral obstruction (UUO) via producing local vasoactive factors leads to increased RVR, decreased RBF, and renal hemodynamic impairment [102, 103]. Hassanshahi and Nematbakhsh showed that Ang 1-7 infusions significantly increased RBF and decreased RVR in sham, UUO, and RUUO (UUO removal) groups [104]. Then examined the role of A779 in response to Ang 1-7 infusion three days after UUO and one day after RUUO [104]. Analysis of the results showed that three days after UUO induction, RBF decreased, and RVR increased in the obstructing kidney but had no effect on MAP. Also, A779 infusion decreased hemodynamic factors [104]. O'Neill et al. showed that the excretory and hemodynamic activities of Ang 1-7 varied at different levels of sodium intake, Mas, and AT1 receptors activity [105]. They observed intrarenal Ang 1-7 infusion in rats on a low-sodium diet increased natriuresis, which was eliminated by Mas and AT1 receptors blockade (losartan and A779) [105]. Therefore, it concluded that natriuresis and diuresis induced by Ang 1-7 depend on the influence of AT1R and the stimulation and nonstimulation of RAS. Finally, the effects of MasR antagonist (A779) in the renal system in different experimental models are listed in Table 2.

## 3. Conclusion

ACE2/Ang 1-7/MasR axis is a vasodilator pathway in RAS, and this pathway's response varies in physiological and pathological conditions and gender. Gaining a more profound knowledge about MasR function in different situations could help develop therapeutic agents for treating hypertension and chronic kidney disease.

## Figures and Tables

**Table 1:** MasR alteration in different physiological and pathological conditions.

Condition/model	Renal MasR expression	Ref	Human/animal
Normotensive	Female > male	[48, 49]	Male and female Sprague-Dawley rats
Normotensive+angiotensin 1-7 infusion	No change	[51]	Male Wistar rats
SHR+angiotensin 1-7 infusion	Decrease	[51]	Male Wistar rats
2K1C	Decrease	[52]	Male Sprague-Dawley rats
SHR+AngII administration	Female > male	[54]	Wistar rats
I/R	Increase	[59, 60]	Male Wistar rats and male C57BL/6 wild-type mice
Diabetic nephropathy	Decrease	[61]	Male C57BL/6 JAX mice
Diabetic nephropathy+AT1R blockade	Increase	[65]	Human

SHR: spontaneously hypertensive rats; 2K1C: two-kidney, one clip hypertensive rats; I/R: renal ischemia/reperfusion.

**Table 2:** The effect of angiotensin 1-7 receptor (MasR) antagonist (A779) in the renal system in different experimental models.

Antagonist MasR (A779)			
Model	Effect		
	Male	Female	Ref
Water loaded male Wistar rats	⬆Urine volume	—	[70]
Virgin female	—	⬆Urine volume	[72]
Pregnant female	—	⬇Urine volume	[72]
Male and female SHRs+Ang II infusion	⬆Blood pressure (there was no sex difference)	⬆Blood pressure (there was no sex difference)	[54]
Male and female Wistar rats+Ang 1-7 infusion	⬇RBF response to Ang 1-7 (the decrease was not significant)	⬇RBF response to Ang 1-7	[88]
Male Wistar rats	⬇RBF	—	[89]
OVX female Wistar rats+ERT	—	⬆RBF response to Ang 1-7 infusion (this response increased significantly in nonestradiol treated compared to the estradiol-treated rats)	[90]
Male and female Wistar rats+block of AT1R+AT2R	⬆RBF response to Ang 1-7 infusion	This response did not occur in female animals	[91, 92]
Male and female Wistar rats+block of AT1R+AT2R	RBF response did not any change between males and females	RBF response did not any change between males and females	[93]
OVX female Wistar rats+BK agonist	—	⬆RBF response to Ang 1-7 infusion (in OVX group was greater than OVX+ERT group insignificantly)	[95]
OVX female Wistar rats+B2R antagonist+ERT	—	⬆RBF response to Ang 1-7 infusion (when B2R alone blocking, the role of estradiol was highlighted)	[96]
Male and female Wistar rats+PD123319	⬇RBF response to Ang II infusion	Attenuated ⬇ RBF response to Ang II infusion in females but not male	[97]
Male and female Wistar rats+2K1C	⬆RBF responses to Ang II infusion (no difference between the sexes)	⬆RBF responses to Ang II infusion (no difference between the sexes)	[98]
Male and female Wistar rats+2K1C+AT1R block	⬇RBF responses to Ang II infusion (this response in males significantly more than in females)	⬇RBF responses to Ang II infusion	[98]
OVX female Wistar rats+ERT	—	⬇RBF response to Ang II infusion (the reduction was significant compared to other groups)	[99]
Female Wistar rats+2K1C+ERT	—	⬇RBF response to Ang II infusion (there was no difference between groups)	[99]
Male Wistar rats+2K1C+IPC+I/R	⬆RBF response to Ang II infusion (significant difference with sham group)	—	[100]
Male Wistar rats+2K1C+I/R	⬆RBF response to Ang II infusion (significant difference with sham group)	—	[100]
Male and female Wistar rats+moderate I/R	⬆RBF response to Ang II infusion (was not significant compared to the vehicle group)	⬆RBF response to Ang II infusion (was significant compared to the vehicle group)	[101]
Male and female Wistar rats+moderate I/R+PD123319	⬆RBF response to Ang II infusion (was not significant compared to the vehicle group)	⬆RBF response to Ang II infusion (was significant compared to the vehicle group)	[101]
Male Wistar rats	⬇RBF response to Ang 1-7 infusion	—	[104]
Male Wistar rats+UUO	⬇RBF response to Ang 1-7 infusion	—	[104]
Male Wistar rats+RUUO	⬇RBF response to Ang 1-7 infusion	—	[104]
Male Wistar rats+normal-sodium diet	Increase GFR, had no significant effect on MAP, UF, UNaV, or FENa	—	[105]
Male Wistar rats+low-sodium diet	No effect on GFR, had no significant effect on MAP, UF, UNaV, or FENa	—	[105]

I/R: ischemia/reperfusion; OVX: ovariectomy; ERT: estrogen replacement therapy; AT1R: angiotensin II type 1 receptor; AT2R: angiotensin II type 2 receptor; BK: bradykinine; B2R: bradykinin B2 receptor; IPC: ischemia preconditioning; RBF: renal blood flow; RVR: renal vascular resistance; Ang II: angiotensin II; Ang 1-7: angiotensin 1-7; UUO: unilateral ureteral obstruction; RUUO: UUO removal; 2K1C: two-kidney, one clip hypertensive model; SHR: spontaneously hypertensive rats.

## Data Availability

No data is available.
